# Reliability and Validity of the Neonatal Pain, Agitation, and Sedation Scale in Mechanically Ventilated Neonates: A Systematic Review

**DOI:** 10.7759/cureus.116730

**Published:** 2026-09-22

**Authors:** Niki Sotiropoulou, Kassiani Theodoraki, Antigoni Sarantaki, Angeliki Bolou, Vikentia Harizopoulou, Athanasia Tsaroucha, Dimitra Metallinou

**Affiliations:** 1 Department of Medicine, National and Kapodistrian University of Athens, Athens, GRC; 2 First Department of Anesthesiology, Aretaieion University Hospital, School of Medicine, National and Kapodistrian University of Athens, Athens, GRC; 3 Department of Midwifery, University of West Attica, Athens, GRC

**Keywords:** mechanical ventilation, neonatal intensive care, neonatal pain, n-pass, reliability, sedation assessment, systematic review, validity

## Abstract

Accurate assessment of pain, agitation, and sedation in mechanically ventilated neonates is fundamental to neonatal intensive care but remains challenging because neonates cannot self-report pain, and behavioral and physiological responses are influenced by illness severity, medication exposure, and developmental maturity. This systematic review evaluated the reliability, validity, and clinical utility of the Neonatal Pain, Agitation and Sedation Scale (N-PASS) in mechanically ventilated neonates. The review was conducted in accordance with the PRISMA 2020 guidelines. PubMed/MEDLINE, Scopus, and CINAHL were systematically searched from each database’s inception through March 2026, with supplementary screening of reference lists. Primary quantitative studies evaluating the N-PASS in mechanically ventilated neonates or reporting separate data for this population were eligible. Owing to methodological heterogeneity, the findings were synthesized narratively, and the risk of bias was assessed using the Joanna Briggs Institute (JBI) Critical Appraisal Checklist for Analytical Cross-Sectional Studies. Four studies conducted in India, China, Iran, and Egypt met the inclusion criteria. The available evidence indicated high to excellent inter-rater reliability and moderate to good internal consistency, while the findings supported the convergent, criterion, and discriminant validity of the N-PASS in mechanically ventilated neonates. Evidence also supports its clinical utility for routine assessment of pain, agitation, and sedation in neonatal intensive care. Nevertheless, the evidence base remains limited and heterogeneous, particularly with respect to the sedation component and the discrimination of intermediate levels of sedation. Further well-designed multi-center studies involving larger and more homogeneous populations are warranted to strengthen the evidence supporting the use of the N-PASS in mechanically ventilated neonates.

## Introduction and background

Hospitalized neonates in the neonatal intensive care unit (NICU), particularly those requiring mechanical ventilation, are frequently exposed to painful and stressful stimuli related to invasive procedures, routine handling, endotracheal suctioning, prolonged respiratory support, and the intensive care environment itself [1-3]. Accurate assessment of pain, agitation, and sedation is therefore essential for optimizing analgesic and sedative therapy, promoting patient-ventilator synchrony, and minimizing adverse clinical outcomes.

Balancing analgesia and sedation in mechanically ventilated neonates remains clinically challenging. Inadequate treatment may result in pain, agitation, physiological instability, and patient-ventilator asynchrony, whereas excessive sedation may reduce spontaneous respiratory effort, prolong mechanical ventilation, and increase the risk of adverse outcomes [4-6]. This balance makes an integrated assessment of pain, agitation, and sedation particularly relevant in mechanically ventilated neonates, as clinical management requires recognition of both insufficient symptom control and excessive sedation. The assessment is further complicated by the inability of neonates to self-report pain and by the influence of gestational age, illness severity, medication exposure, and neurodevelopmental maturity on behavioral and physiological responses.

Although several neonatal pain assessment tools are available, many were developed primarily for acute procedural pain and may be less suitable for evaluating prolonged pain, agitation, or sedation in critically ill mechanically ventilated neonates [7]. The Neonatal Pain, Agitation and Sedation Scale (N-PASS) was developed as an observational tool for the integrated assessment of pain, agitation, and sedation in the neonates requiring intensive care, including those receiving mechanical ventilation or postoperative care [8]. The scale evaluates five behavioral and physiological domains - crying/irritability, behavior/state, facial expression, extremity tone, and vital signs - with each domain contributing to separate pain/agitation and sedation scores. Higher positive scores indicate increasing pain/agitation, whereas increasingly negative scores indicate deeper sedation. This integrated scoring framework allows pain/agitation and sedation to be assessed within the same instrument, making the N-PASS particularly relevant to the clinical challenges associated with mechanical ventilation.

Despite its widespread clinical use, evidence supporting the psychometric performance of the N-PASS in mechanically ventilated neonates remains limited and heterogeneous. Published studies differ in study design, patient characteristics, comparator instruments, and the psychometric properties evaluated, making it difficult to draw definitive conclusions regarding the performance of the scale in this high-risk population [4,9,10]. The established neonatal pain assessment instruments used for comparison include the Premature Infant Pain Profile (PIPP) [11], its revised version (PIPP-R) [12], and the Neonatal Infant Acute Pain Assessment Scale (NIAPAS), against which the aspects of N-PASS validity have been evaluated. Although previous reviews have examined neonatal pain assessment tools or the broader use of the N-PASS, no systematic review has specifically synthesized the available evidence regarding its reliability, validity, and clinical utility exclusively in mechanically ventilated neonates.

## Review

Methods

Study Design

This systematic review was not registered in the International Prospective Register of Systematic Reviews (PROSPERO) and was conducted and reported in accordance with the Preferred Reporting Items for Systematic Reviews and Meta-Analyses (PRISMA) 2020 guidelines [13]. Owing to methodological and clinical heterogeneity across the included studies, findings were synthesized narratively, and no meta-analysis was performed.

Search Strategy

A systematic literature search was conducted in PubMed/MEDLINE, Scopus, and CINAHL from each database's inception through March 2026. Google Scholar was used only for supplementary identification of potentially relevant studies and was not included in the PRISMA record count. The reference lists of all included studies were also screened to identify additional eligible publications.

The search strategy combined four concept blocks related to the N-PASS instrument, neonatal population, mechanical or assisted ventilation, and pain, agitation, sedation, psychometric properties, or clinical utility. Search terms included “N-PASS,” “Neonatal Pain, Agitation and Sedation Scale,” neonate, newborn, preterm, infant, “mechanical ventilation,” “assisted ventilation,” ventilated, intubated, pain, agitation, sedation, reliability, validity, psychometric, and “clinical utility.” Terms within each concept block were combined using the Boolean operator OR, and the four concept blocks were combined using AND. Database-specific field syntax was applied as appropriate. Where available, filters for English-language publications and human subjects were applied. The complete database-specific search strategies for PubMed/MEDLINE, Scopus, and CINAHL, including the Boolean operators and database-specific field syntax, are provided in Appendix A.

Eligibility Criteria

Primary quantitative studies were eligible if they evaluated the psychometric properties or clinical utility of the N-PASS in preterm or term neonates receiving invasive mechanical ventilation via an endotracheal tube. Studies including mixed neonatal populations were eligible only if data for mechanically ventilated neonates were reported separately.

Outcomes of interest included inter-rater reliability, internal consistency, convergent, criterion, and discriminant validity, agreement with other assessment instruments or clinical judgment, and clinical utility.

Reviews, meta-analyses, editorials, letters, study protocols, case reports, and studies without primary research data were excluded. Studies were also excluded if they did not evaluate the psychometric properties or clinical utility of the N-PASS, did not report separate data for mechanically ventilated neonates, were not published in English, had no accessible full text, or provided insufficient methodological information.

Study Selection, Data Extraction, and Risk-of-Bias Assessment

Records were independently screened by two authors according to the predefined eligibility criteria. Titles and abstracts were screened first, followed by independent full-text assessment of potentially eligible studies. Disagreements regarding study eligibility were resolved through discussion and consensus. Study selection followed the PRISMA 2020 process of identification, screening, eligibility assessment, and inclusion.

For each included study, data were extracted by one author and checked for accuracy by a second author. Data extracted included publication year, country, study design, sample characteristics, clinical setting, N-PASS component evaluated, comparator instrument or clinical indicator, and outcomes related to reliability, validity, and clinical utility.

Risk-of-bias assessments were performed by one author and independently checked by a second author, with any discrepancies resolved through discussion and consensus. Risk of bias was assessed using the Joanna Briggs Institute (JBI) Critical Appraisal Checklist for Analytical Cross-Sectional Studies [14]. This tool was selected to provide a structured and consistent assessment of the overall methodological quality of the included observational studies. The assessment focused on participant selection, measurement methods, confounding, and statistical analysis. Because the JBI checklist is not specifically designed for measurement-property studies, the findings were interpreted with this limitation in mind. Domain-level judgments were summarized as low, moderate, unclear, or not applicable.

Results

Study Selection

The study selection process is shown in Figure 1. A total of 88 records were identified through database searching, including PubMed/MEDLINE (n = 38), Scopus (n = 31), and CINAHL (n = 19). After removal of 27 duplicates, 61 records underwent title and abstract screening, of which 43 were excluded. Eighteen full-text articles were assessed for eligibility, and 14 were excluded because they did not meet the predefined eligibility criteria. Ultimately, four studies met the inclusion criteria and were included in the review [6,9,10,15].

**Figure 1:**
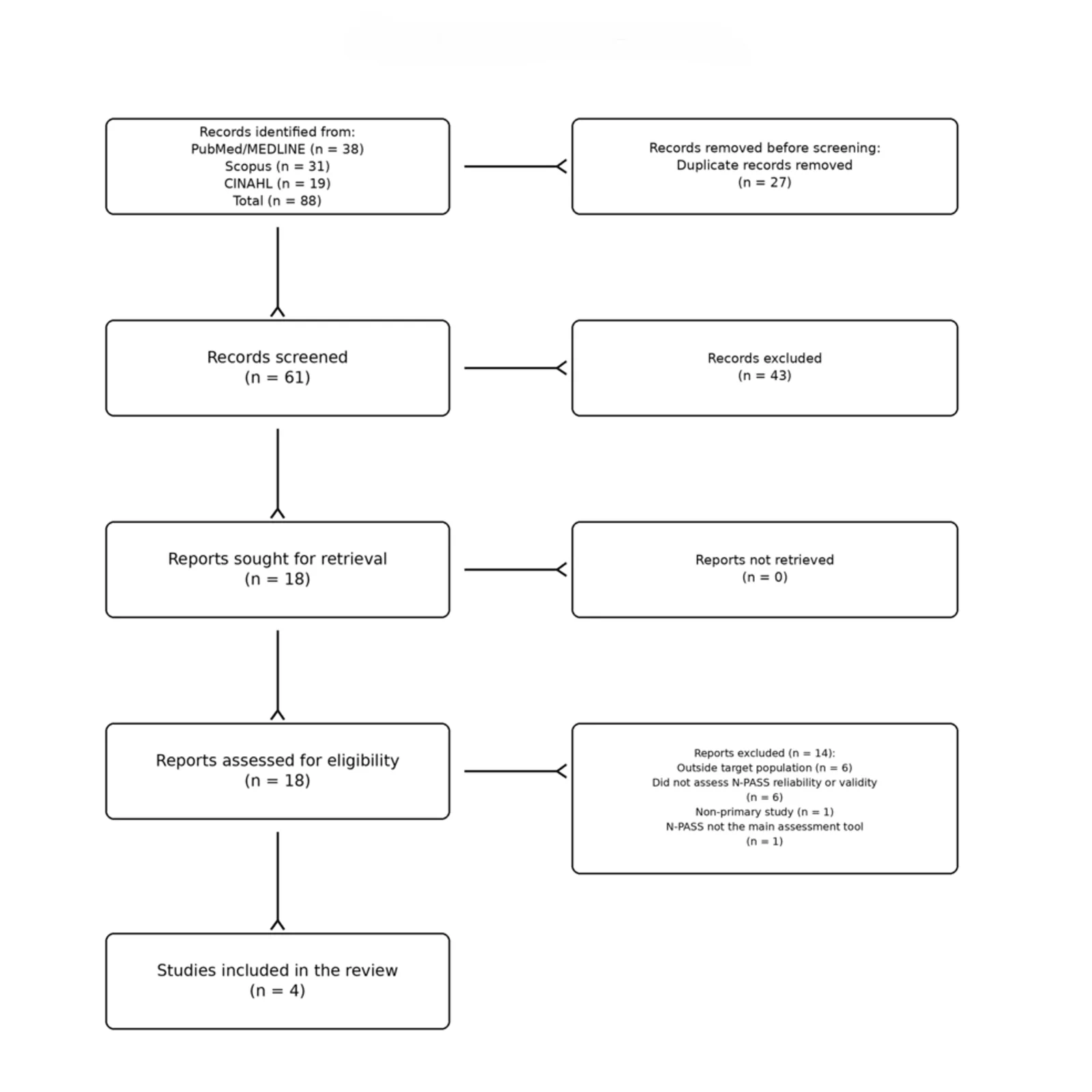
PRISMA 2020 flow diagram of study identification, screening, eligibility assessment, and inclusion. PRISMA: Preferred Reporting Items for Systematic Reviews and Meta-Analyses

Characteristics of Included Studies

The characteristics of the included studies are summarized in Table 1. The studies were conducted in India, China, Iran, and Egypt and used prospective observational, cross-sectional, or methodological/psychometric designs. Sample sizes ranged from 30 to 90 neonates, with some studies including repeated assessments. The included studies evaluated mechanically ventilated neonates either exclusively or as separately reported subgroups within the broader neonatal populations. The N-PASS was evaluated for either pain/agitation or sedation and was compared with the established assessment instruments or clinical indicators, including the PIPP, the PIPP-R, the NIAPAS, and amplitude-integrated electroencephalography (aEEG).

**Table 1:** Characteristics of the included studies. aEEG: amplitude-integrated electroencephalography; PIPP: premature infant pain profile; NIAPAS: Neonatal Infant Acute Pain Assessment Scale; ICC: intraclass correlation coefficient; PIPP-R: premature infant pain profile-revised

Study	Country	Design	Sample	Population	N-PASS use	Comparator	Main psychometric findings
Heidarpour et al. [15]	Iran	Methodological/psychometric	30 intubated neonates (eligible subgroup of a mixed neonatal sample)	Mixed neonatal population; mechanically ventilated subgroup analyzed separately	Sedation	Clinical sedation status	Inter-rater reliability for the sedation component: ICC = 0.88 during intravenous blood sampling and 0.92 during suctioning; internal consistency: Cronbach’s α = 0.79 and 0.66, respectively. Criterion and discriminant validity were supported for sedation assessment.
Ibrahim et al. [6]	Egypt	Cross-sectional	30 neonates	Mechanically ventilated neonates	Sedation	aEEG (Burdjalov score)	N-PASS total scores differed significantly between lightly and deeply sedated neonates (median −3 vs. −7, respectively; p < 0.001), supporting discrimination between sedation levels.
Huang et al. [10]	China	Prospective	90 neonates	Mechanically ventilated neonates	Pain/agitation	PIPP-R, NIAPAS	Inter-rater reliability: ICC = 0.99 (p < 0.001); internal consistency: Cronbach’s α = 0.837. N-PASS correlated with PIPP-R (r = 0.787, p < 0.001) and NIAPAS (r = 0.823, p < 0.001), supporting concurrent validity; discriminant validity was also demonstrated.
Desai et al. [9]	India	Prospective observational	42 neonates (21 invasively ventilated)	Assisted-ventilation population; invasive-ventilation subgroup analyzed separately	Pain/agitation	PIPP	For invasively ventilated neonates, sensitivity = 75%, specificity = 100%, positive predictive value = 100%, and negative predictive value = 50% compared with PIPP. Inter-rater reliability was not assessed.

Reliability

Inter-rater reliability, reflecting the degree of agreement between different assessors, was high to excellent in the studies that assessed it. Heidarpour et al. reported intraclass correlation coefficients (ICCs) of 0.88-0.92 for the sedation component in intubated neonates, while Huang et al. reported an ICC of 0.99 for the pain/agitation component in invasively ventilated neonates [10,15]. Internal consistency, reflecting the extent to which items within the scale assess related aspects of the construct, was assessed in two studies. Among mechanically ventilated neonates, Heidarpour et al. [15] reported Cronbach’s α coefficients of 0.79 and 0.66 for the sedation component during intravenous blood sampling and suctioning, respectively, while Huang et al. [10] reported a Cronbach’s α of 0.837 for the N-PASS, indicating moderate to good internal consistency across the evaluated conditions.

Validity

Evidence supporting the validity of the N-PASS was demonstrated through convergent, criterion, and discriminant validity. Convergent validity, referring to the degree of agreement with other instruments assessing related constructs, was supported by correlations with established neonatal pain assessment instruments. In mechanically ventilated neonates, Huang et al. [10] reported significant correlations between the N-PASS and the PIPP-R (r = 0.787, p < 0.001) and NIAPAS (r = 0.823, p < 0.001). Discriminant validity, reflecting the ability of the scale to distinguish between clinically different states, was supported by the differentiation of clinically distinct pain and sedation states by the N-PASS [6,10,15]. However, available evidence suggests that discrimination between intermediate levels of sedation remains more limited.

Clinical Utility

The available evidence supports the clinical utility of the N-PASS in routine neonatal intensive care practice. The scale was considered practical for bedside use and demonstrated the ability to reflect clinically meaningful changes in neonatal status [10]. Its integrated assessment of pain/agitation and sedation may inform analgesic and sedative management in mechanically ventilated neonates while supporting patient comfort, respiratory drive, and patient-ventilator synchrony [6].

Risk of Bias

Overall, the included studies were judged to have a low to moderate risk of bias (Table 2). The principal methodological limitations included limited control of potential confounding factors, sample heterogeneity, and incomplete reporting in selected domains. Measurement methods and statistical analyses were generally judged to be at low risk of bias. Because risk of bias was assessed using a generic appraisal tool for observational studies rather than an instrument specifically designed for measurement-property studies, these findings should be interpreted as an assessment of overall methodological quality rather than a comprehensive evaluation of psychometric quality. Detailed justifications for the overall risk-of-bias judgments are provided in Appendix B.

**Table 2:** Risk-of-bias assessment of the included studies. The risk-of-bias assessment was based on the JBI Critical Appraisal Checklist for Analytical Cross-Sectional Studies. Domain-level labels summarize item-level judgments. The overall risk-of-bias judgment was based on the combined pattern of ratings across domains, rather than on isolated criteria. JBI: Joanna Briggs Institute

Study	Selection bias	Measurement validity	Exposure validity	Outcome validity	Confounding factors	Statistical analysis	Overall risk of bias
Ibrahim et al. [6]	Moderate	Moderate	Low	Moderate	Moderate	Low	Moderate
Heidarpour et al. [15]	Low	Low	Low	Low	Unclear	Low	Low-to-moderate
Huang et al. [10]	Low	Low	Low	Low	Moderate	Low	Low-to-moderate
Desai et al. [9]	Low	Low	Low	Low	Unclear	Low	Low-to-moderate

Discussion

This systematic review synthesized the available evidence on the reliability, validity, and clinical utility of the N-PASS in mechanically ventilated neonates. Overall, the findings provide support for the reliability, validity, and clinical utility of the N-PASS for assessing pain, agitation, and sedation in this high-risk population. However, these findings should be interpreted with caution because the available evidence remains limited and methodologically heterogeneous. To our knowledge, this is the first systematic review specifically examining the psychometric performance of the N-PASS in mechanically ventilated neonates.

Assessment of pain, agitation, and sedation in mechanically ventilated neonates remains inherently challenging because it relies on behavioral and physiological indicators that are influenced by gestational age, illness severity, medication exposure, and neurodevelopmental maturity [16]. Although several neonatal pain assessment instruments are available, differences in their intended purpose, psychometric performance, and target populations continue to complicate instrument selection in clinical practice [17]. N-PASS is particularly relevant because it was specifically developed to provide integrated assessment of both pain/agitation and sedation, supporting more individualized management of analgesic and sedative therapy in critically ill neonates [8].

A principal finding of this review is the favorable reliability of the N-PASS in the studies that assessed it. High to excellent inter-rater reliability indicates that the instrument can be applied reproducibly by different clinicians, while moderate to good internal consistency supports the coherence of its measurement domains. These findings are consistent with the original validation study by Hummel et al. [8], although that work included a broader neonatal population rather than exclusively mechanically ventilated neonates.

The available evidence also supports the validity of the N-PASS. In mechanically ventilated neonates, correlations with established neonatal pain assessment instruments provide evidence supporting convergent validity, while the ability of the N-PASS to differentiate clinically distinct pain and sedation states supports discriminant validity [6,10,15]. Criterion validity was also supported in the eligible intubated population evaluated by Heidarpour et al. [15]. Additional evidence demonstrating agreement with bedside clinical assessment and treatment decisions further supports the clinical applicability of the N-PASS beyond formal psychometric evaluation [18,19].

The sedation component of the N-PASS warrants particular attention. Although the available evidence indicates that the scale can differentiate levels of sedation, its ability to discriminate between intermediate sedation levels appears more limited than its ability to identify deeper sedation [4,6]. This limitation has important clinical implications because both under-sedation and over-sedation may adversely affect patient comfort, ventilator synchrony, duration of mechanical ventilation, and overall neonatal outcomes [20]. Further refinement and validation of the sedation component may therefore enhance the clinical utility of the instrument.

This review has several strengths. By focusing specifically on mechanically ventilated neonates, it addresses a clinically important population that has received limited attention in previous evidence syntheses. The review also applied a systematic search strategy across multiple databases, followed PRISMA 2020 recommendations, and incorporated structured risk-of-bias assessment. Nevertheless, several limitations should be acknowledged. Only four studies met the eligibility criteria, and although the search was supplemented by Google Scholar and reference-list screening, it was limited to three bibliographic databases and did not include Embase or a formal grey literature search. Additional eligible studies may therefore have been missed. Moreover, substantial heterogeneity in study design, sample characteristics, comparator instruments, and reported psychometric outcomes precluded quantitative synthesis. Accordingly, the findings should be interpreted as a narrative synthesis rather than definitive estimates of the measurement properties of the N-PASS. Risk of bias was assessed using the JBI Critical Appraisal Checklist for Analytical Cross-Sectional Studies; however, as this tool was not specifically developed for measurement-property studies, certain aspects relevant to psychometric research may not have been fully captured.

Overall, the available evidence supports the reliability, validity, and clinical utility of the N-PASS for assessing pain, agitation, and sedation in mechanically ventilated neonates. Nevertheless, the limited number of available studies and their methodological heterogeneity highlight the need for further well-designed multicenter research involving larger and more homogeneous populations. Future studies should particularly focus on strengthening the evidence supporting the sedation component of the scale, clarifying its ability to distinguish intermediate levels of sedation, and evaluating its performance across specific neonatal subgroups.

## Conclusions

This systematic review provides support for the reliability, validity, and clinical utility of the N-PASS for assessing pain, agitation, and sedation in mechanically ventilated neonates. The available evidence suggests that it may serve as a structured bedside assessment tool to support pain and sedation assessment and inform analgesic and sedative management in neonatal intensive care.

Nevertheless, the current evidence base remains limited by the small number of available studies and substantial methodological heterogeneity. Additional well-designed multi-center studies involving larger and more homogeneous populations are needed to further establish the psychometric performance of the N-PASS, particularly regarding the assessment of intermediate levels of sedation and its application across different neonatal subgroups.

## Appendices

Appendix A 

**Table 3:** Database-specific electronic search strategies.

PubMed/MEDLINE	
Item	Search strategy
Final search	("N-PASS" OR "Neonatal Pain, Agitation and Sedation Scale") AND (neonate OR newborn OR preterm OR infant) AND ("mechanical ventilation" OR "assisted ventilation" OR ventilated OR intubated) AND (pain OR agitation OR sedation OR reliability OR validity OR psychometric OR "clinical utility")
Scopus	
Item	Search strategy
Final search	TITLE-ABS-KEY ("N-PASS" OR "Neonatal Pain, Agitation and Sedation Scale") AND TITLE-ABS-KEY (neonate OR newborn OR preterm OR infant) AND TITLE-ABS-KEY ("mechanical ventilation" OR "assisted ventilation" OR ventilated OR intubated) AND TITLE-ABS-KEY (pain OR agitation OR sedation OR reliability OR validity OR psychometric OR "clinical utility")
CINAHL (EBSCOhost)	
Search line	CINAHL search strategy
S1	TX "N-PASS" OR TX "Neonatal Pain, Agitation and Sedation Scale"
S2	TX neonate OR TX newborn OR TX preterm OR TX infant
S3	TX "mechanical ventilation" OR TX "assisted ventilation" OR TX ventilated OR TX intubated
S4	TX pain OR TX agitation OR TX sedation OR TX reliability OR TX validity OR TX psychometric OR TX "clinical utility"
S5	S1 AND S2 AND S3 AND S4

Appendix B

**Table 4:** Rationale for overall risk-of-bias judgments. This appendix summarizes the rationale for the overall risk-of-bias judgments reported in Table 2.

Study	Rationale
Ibrahim et al. [6]	Moderate overall risk because of limited control of confounding and moderate concerns regarding measurement and outcome validity, despite appropriate statistical analysis.
Heidarpour et al. [15]	Low-to-moderate overall risk because most methodological domains were clearly described and judged adequate, while confounding remained unclear.
Huang et al. [10]	Low-to-moderate overall risk because measurement and analysis were appropriate, but potential confounding and sample heterogeneity were not fully addressed.
Desai et al. [9]	Low-to-moderate overall risk because core measurement domains were adequate, but reporting around confounding was limited.
